# Content Validity Testing of a Nurse-Led Pediatric Dysphagia Screening Tool for Acute and Critical Care Settings Using eDelphi Methodology

**DOI:** 10.3390/children12121626

**Published:** 2025-11-30

**Authors:** Christie Grunke, Elizabeth C. Ward, Anna Miles, Bronwyn Carrigg, Sainath Raman, Loretta Scaini, Louise Edwards, Memorie M. Gosa, Jeanne Marshall

**Affiliations:** 1 School of Health and Rehabilitation Sciences, The University of Queensland, Brisbane 4072, Australia; christie.grunke@student.uq.edu.au (C.G.);; 2 Speech Pathology Department, Queensland Children’s Hospital, Brisbane 4101, Australia; 3 Centre for Functioning and Health Research (CFAHR), Metro South Hospital and Health Service, Brisbane 4102, Australia; 4 Department of Speech Science, School of Psychology, The University of Auckland, Auckland 1010, New Zealand; 5 The Royal Children’s Hospital, Melbourne 3052, Australia; 6 Children’s Intensive Care Research Program, Child Health Research Centre, The University of Queensland, Brisbane 4072, Australia; 7 Paediatric Intensive Care Unit, Queensland Children’s Hospital, Brisbane 4101, Australia; 8 Leeds Teaching Hospitals National Health Service Trust, Leeds LS1 3EX, UK; 9 School of Community Health and Midwifery, University of Lancashire, Preston PR1 7QS, UK; 10 Department of Communicative Disorders, The University of Alabama, Tuscaloosa, AL 35487, USA

**Keywords:** deglutition disorders, risk factors, pediatrics, patient care, mass screening

## Abstract

**Highlights:**

**What are the main findings?**

- Eight clinical presentations, three oral trial elements, three oral trial protocols, and fourteen signs suggestive of oropharyngeal dysphagia were agreed on by panelists and the steering group to include in the novel Children’s Oral Feeding Screener (COFS) tool.
- The COFS is positioned as one of the first nurse-led pediatric dysphagia screening tools designed broadly for all children admitted to acute and/or critical care settings, which contains a structured fluid oral trial component spanning typical pediatric intake methods.

**What is the implication of the main finding?**

- Initial content validity for three components of the COFS (red flags, oral trial elements/protocols, and observable signs of dysphagia) for use in children admitted to acute and/or critical care hospital settings has been developed through this eDelphi process.
- Further psychometric testing of the construct validity, sensitivity/specificity, inter-rater reliability, and feasibility of this tool in these settings remains to be established in future studies.

**Abstract:**

**Background**: Pediatric oropharyngeal dysphagia screening protocols remain limited in acute and critical care settings due to the lack of psychometrically valid and reliable tools. **Objectives:** The eDelphi methodology was employed to establish content validity for the Children’s Oral Feeding Screener (COFS), a novel, nurse-led oropharyngeal dysphagia screening tool for hospitalized children in acute and critical care (0–16 years). **Methods**: The two-round eDelphi study was completed using Qualtrics^®^. A multidisciplinary, international steering-group guided tool conceptualization, elements for rating in the eDelphi, and oversaw consensus decisions. Experienced speech pathologists in pediatric acute and/or critical care were invited as panelists and rated tool content regarding (a) clinical presentations requiring immediate referral for dysphagia assessment; (b) oral trial component/s; and (c) signs observed during oral trials suggesting dysphagia. Items were rated on a 10-point Likert scale, and panelists could give open-ended feedback. Items not reaching pre-defined consensus (>75%) were re-presented in round two. **Results**: Fifty panelists participated in round one and 41 in round two, primarily from Australia (n = 19; 46%) and the United Kingdom (n = 13; 34%). Half (n = 22; 54%) had >10 years’ experience. Based on consensus scores, panelists’ qualitative feedback, and steering group decision, final items included eight clinical presentations, three oral trial elements (cup, bottle, breastfeeding) with three associated oral trial protocols, and fourteen signs suggestive of dysphagia. Other feedback led to changes to headings and the format of the COFS layout. **Conclusions**: Content validity for items in the three components of the COFS was established. Further work is now required to explore other psychometric properties (construct validity, sensitivity/specificity, and feasibility) in clinical settings.

## 1. Introduction

Oropharyngeal dysphagia (herein referred to as “dysphagia”) describes difficulties in the oral and pharyngeal phases of swallowing [1]. In children, dysphagia may be transient, developmental, chronic, and/or progressive in nature [2] and may occur due to several factors, including prematurity, congenital or genetic abnormalities, neurological impairments, and structural abnormalities [3,4,5]. For children hospitalized in acute and critical care settings, the prevalence of dysphagia has been reported to be as high as 91% in children with neurological conditions and 74% in children with cardiac conditions [6]. These prevalence figures are notably higher in children who are younger, or who have lower weight, upper/middle airway dysfunction, and/or greater length of intubation [6].

Dysphagia poses significant health risks for children, including aspiration pneumonia, choking, dehydration, and poor weight gain [7,8]. It can also significantly impact the quality of life for affected children and families, cause financial burden through increased specialized feeding equipment needs, and increase hospital and intensive care unit admissions [9,10,11,12,13]. Hence, early identification of dysphagia is paramount to minimize these potential health, quality of life, and financial impacts [4].

Early identification of children at risk of dysphagia can be achieved through dysphagia screening [14,15,16,17,18]. Screening is considered to be the first step in a comprehensive dysphagia assessment [19]. Screening does not diagnose dysphagia, but rather identifies those who are at risk who require a comprehensive swallow evaluation by a dysphagia specialist (e.g., speech pathologist) [19]. In hospital settings, dysphagia screening is often completed by nursing staff in the first instance [19]. Efficacy for nurse-led dysphagia screening in hospital settings is well documented for adult populations such as acute stroke [15,16,18,20], with reductions in aspiration pneumonia rates, intensive care unit and hospital length of stays, and economic burden reported [15,16,20,21,22]. Unfortunately, at present, there are limited published pediatric dysphagia screening tools or guidelines to support members of the multidisciplinary team to screen for dysphagia in this population [23]. Existing pediatric tools are either intended for populations with chronic dysphagia in outpatient settings (e.g., cerebral palsy) and/or are purely based on parent report (The Pediatric Version of the Eating Assessment Tool [PEDI-EAT-10] and The Screening Tool of Feeding Problems Applied to Children [STEP-CHILD]) [24,25]. A handful of screening tools for acute and critical care hospitalized children do exist, but these are either in their early content validation phase and have not yet been published [21], validated for single specific populations (e.g., children with congenital heart disease) (The Infant Malnutrition and Feeding Checklist for Congenital Heart Disease [IMFC:CHD]) [26], specific ages (e.g., >2 years old—The 3-ounce Water Swallow Challenge) [27,28], specific oral intake methods (e.g., sipper/open cup drinking) (The 3-ounce Water Swallow Challenge and the Readiness, Oral Skills, Safe, Efficient [ROSE] Feeding Checklist) [27,28,29] and/or do not contain an oral trial component (The Pediatric Screening-Priority Evaluation Dysphagia [PS-PED] and the Pediatric Dysphagia Risk Screening Instrument [PDRSI]) [23,30].

Whilst valid and reliable general dysphagia screening tools for adults do exist, and certain aspects can be adapted for some pediatric populations, exact extrapolation of these tools is not possible. This is due to several factors, including challenges with asking children to follow commands or consume specified volumes, and differences in how dysphagia presents in children (i.e., eating and drinking skills may still be developing) [7]. Additionally, the oral trial components in adult dysphagia screening tools have not undergone full psychometric testing in pediatric populations, and there is currently no published oral trial protocol accommodating the full range of pediatric intake methods (e.g., breast, bottle, sipper cup, and progression of solid textures). As such, there remains a gap in available nurse-led pediatric dysphagia screening tools with proven reliability and accuracy [21] that meet the needs of hospitalized children.

Recognizing the lack of appropriate tools for conducting inpatient pediatric dysphagia screening, the Children’s Oral Feeding Screener (COFS) was developed as a nurse-led dysphagia screening tool, designed to facilitate dysphagia referrals for children at risk of dysphagia admitted to acute and/or critical care hospital settings. The COFS is positioned as one of the first nurse-led pediatric dysphagia screening tools designed broadly for all children admitted to acute and/or critical care settings, which contains a structured fluid oral trial component spanning typical pediatric intake methods. By screening swallow readiness and safety, the COFS aims to guide decision-making regarding progressing oral feeding versus referral to a dysphagia specialist (e.g., speech pathologist) for a comprehensive swallow assessment. The aim of the current study was to formally establish the content validity of the test items within the COFS using subject matter expert consensus via an eDelphi process guided by the Consensus-based Standards for the Selection of Health Measurement Instruments (COSMIN) framework [31]. The specific objectives of the eDelphi were to gather expert consensus on the content of three components of the COFS tool: (a) clinical presentations requiring immediate referral for further dysphagia assessment; (b) oral trial component/s; and (c) clinical signs observed during oral trials suggestive of dysphagia.

## 2. Materials and Methods

### 2.1. Study Design

The eDelphi approach was chosen for this research to help gather expert consensus on best practices and achieve agreement on essential items to be included in the proposed COFS tool [32]. Study design was guided by published literature and recommendations for conducting Delphi and eDelphi studies [32,33,34]. Reporting of this study was guided by the Delphi studies in social and health sciences—recommendations for an interdisciplinary standardized reporting (DELPHISTAR) guidelines [35] (Table A1).

This eDelphi study was guided by a multidisciplinary steering group with international representation from speech pathology (Australia n = 3; New Zealand n = 1; United States of America n = 1; United Kingdom n = 1) as well as members from medicine (n = 1, Australia) and nursing (n = 1, Australia). Members of the steering group were all experienced practitioners (>five years clinical experience with pediatric dysphagia) and/or experienced researchers in the field of pediatric dysphagia and acute and critical care hospital environments. The steering group was convened alongside the author group to consult on eDelphi methodology, support initial conceptualization of an original layout of the tool, and support dissemination of the eDelphi internationally. This steering group also advised on the wording of items to meet the needs of international end-users and resolved final items that did not achieve consensus at the conclusion of two eDelphi rounds [36,37]. This group used email communication and met virtually prior to commencement of the eDelphi and after each round to interpret results [36].

### 2.2. Ethical Approval

The Human Research Ethics Committee at The University of Queensland granted approval for this project on 25 June 2024 (project number 2024/HE000811).

### 2.3. Development of the COFS Tool

The initial version of the screening tool was developed prior to the current study and was based on a review of the literature and informed by the clinical expertise of a group of five experienced speech pathologists and a pediatric intensive care unit (PICU) nurse educator from a tertiary children’s hospital in Australia. That initial tool design then underwent further refinement by the current author group (seven speech pathologists, one PICU nurse educator and one PICU consultant intensivist), incorporating newly published research in pediatric dysphagia in acute and critical care settings [6], learnings from adult dysphagia screening models, and data from a quality improvement trial of the initial version of the COFS within the setting where it was first developed.

**Figure 1:**
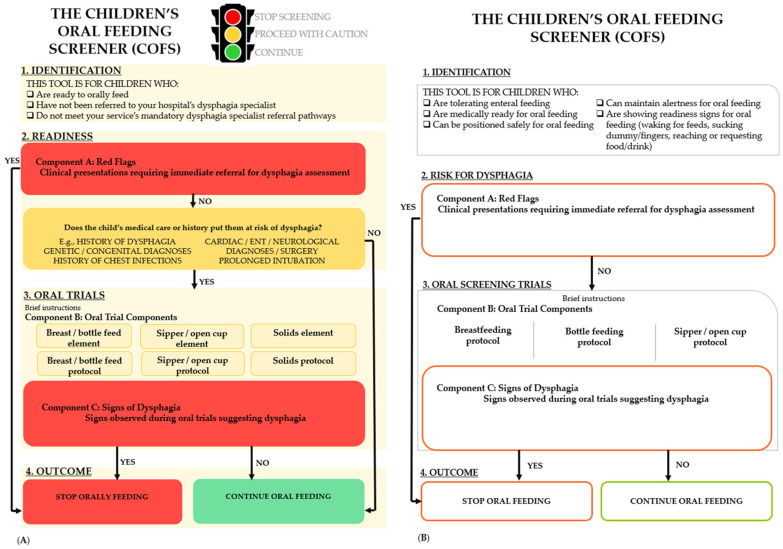
Outline of the Children’s Oral Feeding Screener presented to panelists in (**A**) eDelphi round one and (**B**) post eDelphi round two.

The COFS is structured to function as a flowchart, guiding nursing staff through a series of yes/no decision points to support decision-making as to whether to continue oral feeding or seek further specialist dysphagia assessment. Its drafted outline, as presented to panelists in eDelphi round one (Figure 1A), consisted of four main sections, color-coded according to a traffic light system, red indicating to stop screening, amber to proceed with caution, and green to continue oral feeding. Nursing staff begin in section one “Identification”, which contains instructions identifying who is appropriate for COFS screening. Following identification, advancement through the tool is conditional on presentation, with each subsequent section serving as a safety net for children, where if concerns are flagged, nursing staff are directed to stop and consider referral for further comprehensive swallow assessment. The second section, “Readiness”, contained two boxes: the first box, colored red, was intended to identify red flags with high risk for dysphagia; the second box, colored amber, highlighted past medical history that would place a child at increased risk for dysphagia. If there were no red flags, but the child’s medical history indicated increased risk for dysphagia, then nursing would continue through to the next section, “Oral Trials”. This third section was again split into two boxes: the first, colored amber, was for oral trial elements and protocols to be listed (e.g., breast/bottle feeding, sipper/open cup, and/or solids and their specific protocols); the second box, colored red, was intended to list signs, identifiable to nursing staff, suggestive of dysphagia. The final section, “Outcome”, consisted of two choices pending how nursing progressed through the flowchart: either continue oral feeding or stop and refer for a comprehensive swallow assessment. As shown in Figure 1, three boxes, one in the readiness section “Component A: Red Flags” and two in the oral trial sections “Component B: Oral Trial Components” and “Component C: Signs of Dysphagia”, required input from panelists as to which items to include to support this decision-making process for nursing staff. The “Identification” and “Medical History” boxes were pre-populated by the author group based on the findings from our previously published scoping review [6].

### 2.4. Participants and Recruitment

This study was conducted between June and October 2024. Speech pathologists and occupational therapists with experience assessing and managing dysphagia in pediatric acute and/or critical care settings were identified as eligible panelists for recruitment in this eDelphi study. Inclusion and exclusion criteria are outlined in Table 1.

Qualtrics^®^ software was used to capture information from panelists. Eligible eDelphi panelists were contacted by the author group through an “Email of Invitation”. This invitation was sent to professional and research networks requesting participation in this study (e.g., dysphagia groups and organizations, social media, and professional networks). Snowballing sampling was encouraged. The invitation email contained a link to the initial consent and demographic survey, where panelists created their own unique identifier and provided their email address. Interested clinicians were required to read the participant information sheet, provide digital consent, and complete a brief demographic survey to confirm eligibility for recruitment prior to commencement of the eDelphi rounds. Once recruited to this study, panelists were contacted via their provided email address with links to subsequent eDelphi rounds.

### 2.5. eDelphi Design and Content

The eDelphi survey was conducted in English. All core elements of a classic eDelphi approach were maintained, including preserving anonymity of panelists, use of a standardized questionnaire, and statistical analysis and feedback of results to panelists in subsequent rounds [35]. A modified, two-round eDelphi was employed, with the number of rounds defined in advance to minimize attrition, and on the basis that round one items were pre-populated via the author group’s review of published research, the clinical expertise of the authors and steering group, and by the clinicians who developed earlier versions of the COFS tool [32,34].

In the round one survey, panelists received a demographic summary of the consenting panelists and were reminded of the purpose of the proposed COFS tool. They were then provided with a visual representation of the proposed layout of the COFS tool with the three empty boxes, highlighting the three components they were providing input to for context (Figure 1A). This was followed by three eDelphi sections, one relating to each component with items for rating: (a) “red flags” i.e., clinical presentations requiring immediate referral for a comprehensive swallow assessment by a dysphagia specialist; (b) “oral trials” i.e., nurse-led oral trial screening elements and protocol/s and; (c) “signs of dysphagia” i.e., overt signs identifiable to nursing staff suggestive of dysphagia in pediatrics, requiring immediate referral for a comprehensive swallow assessment by a dysphagia specialist. In round one, panelists were also asked to document if their hospital had any mandatory speech pathology referral pathways for dysphagia assessment in specific pediatric populations (e.g., posterior fossa tumor resections). The online format and design of the eDelphi survey content were first created by the lead author (CG) and then piloted by the author and steering group for functionality, readability, and relevance of questions [37].

In round two of the eDelphi study, panelists were provided with a revised layout of the COFS tool reflecting feedback obtained from round one. Then, in each of the three components, items were categorized under new subheadings: (a) items not for rating, which included those that met consensus for inclusion or exclusion in round one and were provided for reference information only, then (b) items for rating, which included those which failed to be either included or excluded in round one, any reworded items, and any new items for rating. Where items had been reworded for round two based on round one free-text comments and/or subsequent steering group discussions, the changes in wording were displayed alongside previous feedback and original wording to ensure clarity for panelists. In round two, feedback on the round one group response (median and interquartile range) for each item was also graphically provided, so that panelists could see this when re-rating [34].

### 2.6. eDelphi Rounds and Analysis

**Figure 2:**
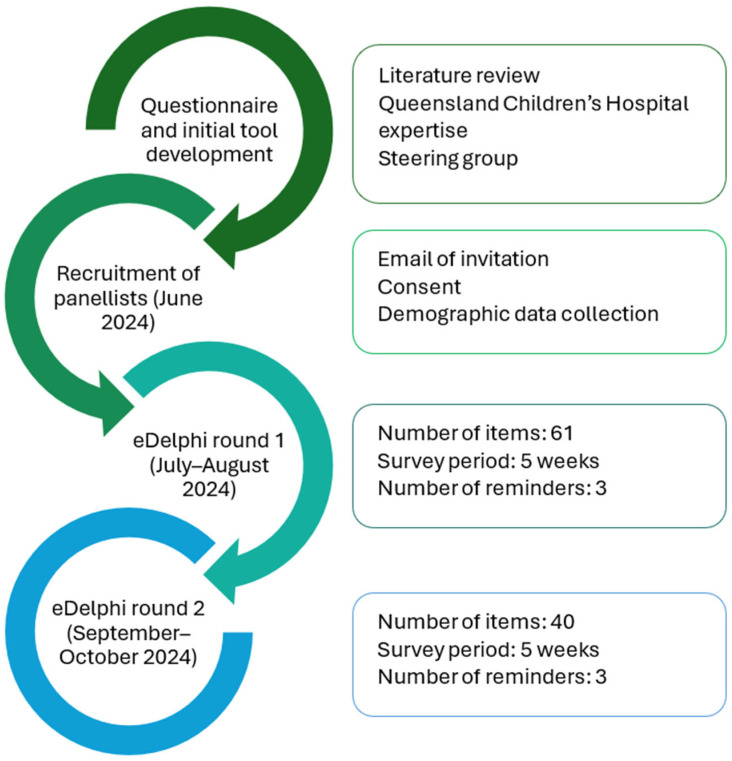
eDelphi process.

The eDelphi was open for approximately five weeks for each round, with two weeks between rounds to analyze results [32] (Figure 2). A maximum of three reminders were sent to panelists to support completion, as well as individual emails expressing gratitude after each round. All recruited panelists were invited to the first eDelphi round, and only those who completed round one were invited to participate in round two.

In both rounds, panelists were asked to rate their agreement that an item should be included in the COFS using a 10-point Likert scale (1 = strongly disagree to 10 = strongly agree) [32,34]. This scale size was chosen to enhance response variability and improve the reliability of items reaching consensus for inclusion [38]. A midpoint was intentionally excluded from the scale to ensure panelists made a positive or negative decision about each statement [34]. An option to provide free text was offered after each part and at the end of the survey for the generation of new items, proposal of modified terminology for items, and development of the visual layout.

In each round, two phases of decisions were made. First, responses to items in each round were analyzed for consensus, which was defined a priori. Consensus was achieved where items scored by >75% of panelists as 1–3 (strongly disagree) were excluded, and those scored by >75% as 8–10 (strongly agree) were included in the final tool, aligning with prior Delphi work in measurement development and COSMIN guidance [33,35]. Second, free text responses were grouped according to the item they referred to (e.g., rewording of an old item, need for a new item, removal of an item) and presented to the steering group to inform any further modifications to items and the COFS tool. At the end of round two, items not reaching the pre-defined consensus thresholds and all new items were presented to the steering group for final decision-making.

## 3. Results

### 3.1. Demographics

In total, 72 panelists completed the consent and demographic survey. Of these, 50 panelists completed eDelphi round one and 41 panelists completed eDelphi round two (82% retention rate between rounds). Panelists who completed both eDelphi rounds were predominantly from Australia (n = 19; 46%) and the United Kingdom (n = 14; 34%), and over half of the panelists had greater than 10 years’ experience (n = 22; 54%). All eDelphi panelists were speech pathologists (n = 72; 100%) (Table 2).

### 3.2. COFS Component A: “Red Flags”

**Figure 3:**
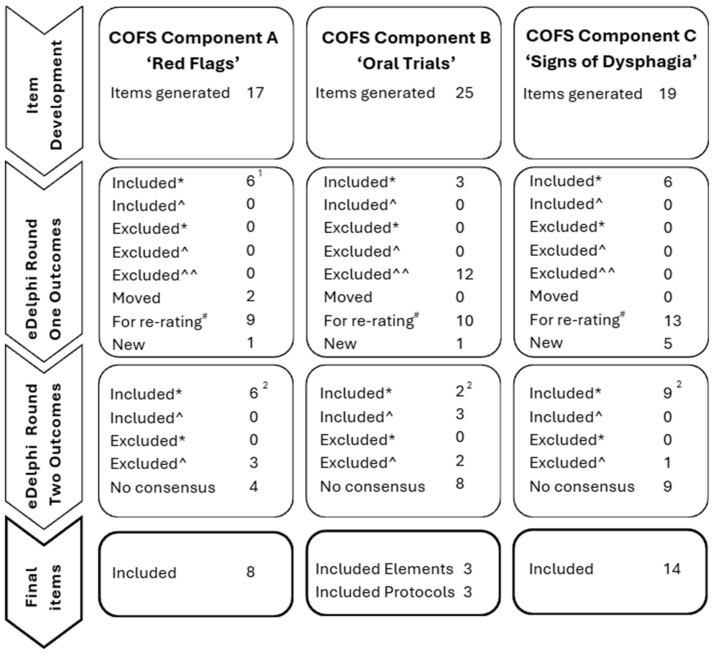
eDelphi decision-making flow chart. Note: * = based on quantitative consensus (>75% agreement), ^ = based on qualitative feedback, ^^ = based on steering group decision, ^#^ = based on quantitative consensus (>25%–<75% agreement), ^1^ = Two items grouped together, ^2^ = Despite reaching >75% agreement for inclusion, some items were later excluded based on qualitative feedback (number later excluded is represented in the ‘Excluded ^’ category).

In round one, 17 potential items for inclusion in the “Red Flags” section were presented to panelists for rating. From these items, six reached quantitative consensus (>75% agreement) for inclusion (new tracheostomy, bilateral vocal fold palsy, unilateral vocal fold palsy, inability to manage secretions, absent cry/voice, absent cough) (Figure 3; Table 3). ‘Absent cough’ was grouped together with ‘weak cough’ based on qualitative feedback and re-presented in round two (Figure 3). No items reached a quantitative consensus to be excluded (Figure 3). Based on qualitative feedback, two items, ‘unable to be positioned in bed safely’ and ‘inability to maintain alertness’, were moved from this component into the identification section at the top of the COFS (Figure 1B). Seven items received rewording suggestions from panelists, which were all agreed on by the steering group. Most of these rewording suggestions were to make terminology more specific and user-friendly. A large number (>50) of new items were suggested by panelists, which included approximately 14 presentations (e.g., acute weight loss, absent gag). Only one of these suggestions was included in round two as a ‘Red Flag’, as agreed upon by the steering group (Figure 3). All other suggested items were removed due to being encompassed by broader items or not indicating an immediate referral to a dysphagia specialist. Furthermore, in the free text, panelists suggested clustering items to indicate the need for direct referral, rather than any one of these presentations alone. Approximately 43 past medical histories (e.g., seizures, spinal muscular atrophy) were also listed by panelists. These were not included as the preparatory scoping review [6] determined the scope of possible medical diagnoses too infinite to be individually listed. Reflecting on qualitative feedback, the “Readiness” and “Oral Trials” sections were modified by the steering group to read “Risk for Dysphagia” and “Oral Screening Trials” (Figure 1B).

Following round two, a further six items reached quantitative consensus (>75%) amongst panelists to be included in the COFS tool (inability to protect airway (e.g., absent or diminished cough), new onset facial asymmetry, parent or nursing report of feeding and/or swallowing difficulties, high flow nasal canula, new onset stridor and unable to move tongue in a controlled way) (Figure 3); however, on review of panelists’ qualitative feedback and discussions amongst the steering group, the final three of these items listed above were subsequently excluded (Table 4). No items reached consensus to be excluded in round two. One item received rewording suggestions. Four items did not reach consensus to include or exclude by panelists and were subsequently excluded during steering group review (Figure 3, Table 4). Qualitative feedback was provided by panelists on the ambiguity of the second ‘medical history’ box in this section of the tool (Figure 1). Based on this and the steering group discussion, this box was removed from the COFS (Figure 1B).

### 3.3. COFS Component B: “Oral Trial Components”

In round one, 25 items were presented to panelists: 15 oral screening elements and 10 oral screening protocols (Figure 3). Three oral screening elements met quantitative consensus for inclusion (Table 3). No elements or protocols met quantitative consensus to be excluded; however, following round one, the steering group agreed to remove all remaining oral screening elements (Figure 3), as these were designed to elicit what panelists felt were important components when creating oral trial protocols for round two. Only one new bottle-feeding oral screening protocol was added to align with suggested breastfeeding protocols (Figure 3). Although panelists provided other protocols that could be included, these were reviewed by the steering committee and determined to be unsuitable to include in round two, as they were comprehensive swallow assessments, rather than screening protocols that could be conducted by untrained users. Based on round one qualitative feedback, references were added to all oral trial protocols that were later included in round two.

Following round two, one bottle-feeding protocol and one solid feeding protocol reached a quantitative consensus to be included (Figure 3; Table 3). Based on qualitative feedback and further steering group discussion, however, the bottle-feeding protocol was subsequently excluded as it represented a full feed observation, rather than an oral trial screen. The solids protocol was also excluded as consensus was not achieved amongst panelists to include observation of solids in the COFS (n = 19; 46%) (Table 4). On consideration of qualitative feedback, high levels of agreement from panelists and existing evidence, three oral trial elements (breastfeeding, bottle feeding and sipper/open cup drinking) and three associated protocols (The 3-ounce Water Swallow Challenge, breastfeed in usual position [observation of at least 3 min] and bottle feed in infants usual position using usual bottle or if not available hospital slow flow teat [observation of at least 3 min]) were selected by the steering group for inclusion in the COFS tool (Figure 3, Table 3), despite not reaching the formal consensus threshold. No items reached a quantitative consensus for exclusion by panelists. Eight items did not reach consensus among panelists and were subsequently excluded on steering group review (Figure 3, Table 4).

In the free text, panelists discussed their hesitations in choosing an oral screening protocol, including the use of unvalidated protocols and the impracticalities of full oral feed observation. There were also concerns regarding nursing staff skills in completing the oral trial protocols, as well as the overall compliance of children.

### 3.4. COFS Component C: “Signs of Dysphagia”

In round one, 19 items were presented to panelists for rating. Six of these items reached a quantitative consensus to be included in the ‘signs of dysphagia’ component of the COFS tool (wet/gurgly breathing, wet vocalizations, apnea with feeding, color changes, oxygen desaturations with feeding, and coughing) (Figure 3; Table 3). No items reached consensus to be excluded, and 10 items received rewording suggestions. Five new items were suggested by panelists to be included in this component of the tool. In the free text, panelists commented on the possibility of a test/re-test scenario to avoid unnecessary referrals for food refusal, avoiding ‘pass/fail’ terminology, and if different signs might indicate the need to ‘stop oral feeding’ depending on the age of the child. It was also suggested to remove background coloring from the tool and instead color the outline of each component for increased readability (Figure 1B).

Following round two, a further eight items reached consensus among panelists to be included (increased respiratory rate/work of breathing outside of child’s typical ranges, new onset or increased wheezing whilst feeding, new onset or increased stridor whilst feeding, fremitus/rattly chest during/after eating or drinking, pooling of food/fluids in child’s mouth that is increased or unusual for them, choking with feeding, child reported pain, or differences to eating or drinking and parental concern with feeding) (Table 3). A ninth item also reached consensus to include (distress cues during oral feeding); however, it was excluded by the steering group based on analysis of the qualitative feedback, who noted this may lead to unnecessary referral. The remaining nine items did not reach consensus among panelists and were subsequently excluded on steering group review (Table 4). In the free text, panelists provided additional comments on the difficulty for nursing staff to assess suck-swallow ratios and the normality of gagging and food refusal in these populations. Final comments by panelists on the overall COFS tool included ‘the need for the tool to be as simple and straightforward as possible’, potential to ‘link the tool to nursing notes so it’s not in another system’, the need for nursing staff training, and some feedback on the readability (font, layout of the tool, images, colors). Overall, there was largely positive feedback on the possibilities of the tool in panelists’ settings.

### 3.5. Mandatory Referral Pathways

Approximately half (n = 26; 52%) of the panelists who participated in round one reported that their hospital had mandatory speech pathology referral pathways. Greater than 100 mandatory pathways were documented as either occurring or incorporated populations that panelists would like to see included. These populations were extremely varied and included any kind of neurological change/injury, head/neck injury or surgery, cardiac surgery, children post extubation, children with a tracheostomy, children on high flow nasal canula, children with a cleft lip and/or palate, and all children on the PICU.

## 4. Discussion

Through a combination of the eDelphi study and input from the expert steering committee, agreement was achieved on key items to be included within the COFS. Ultimately, eight items in the “Red Flags” component, three oral trial elements and three oral trial protocols in the “Oral Trials” component, and 14 signs suggestive of dysphagia in the “Signs of Dysphagia” component (Figure 3, Table 3) were agreed on by panelists and the steering group to be included in the COFS tool. Panelists also aided the development of the overall structure and flow of the COFS to create a clear, logical, and practical tool for end users.

The COFS represents one of the first known nurse-led pediatric dysphagia screening tools designed broadly for all children in acute and/or critical care hospital settings, with a structured fluid oral trial component spanning typical pediatric intake methods. There was strong engagement from panelists, who provided largely positive feedback and supported the need for the development of such a tool. The panel had a high retention rate and included speech pathologists from six different countries, many of whom had over ten years of clinical experience in the field. This global representation, combined with the depth of expertise, contributes to the strength and relevance of the consensus achieved and ultimately the content validity of the items within the tool.

In Component A: “Red Flags”, there was strong agreement amongst panelists, with two-thirds of items reaching consensus after only one eDelphi round (Table 3). Many of these items that reached immediate consensus are known to be associated with dysphagia both in adults and pediatrics, including tracheostomy placement [43], unilateral and bilateral vocal fold palsy [40]. Likely due to this well-established association, these are also items present in many existing pediatric and adult dysphagia screening tools [23,26,29,30,44]. Other items that reached consensus are less researched with regard to their association with pediatric dysphagia, particularly in acute and critical care settings; however, they are still noted as ‘red flags’ for dysphagia in textbooks (e.g., poor secretion management, absent/diminished cough) [7]. These items are generally less common in currently available dysphagia screening tools. However, a recent study that used expert consensus to generate items for a pediatric dysphagia screening tool for use post-acute stroke rated ‘salivary control’ as an item to retain [21], despite weak evidence. This further supports that items from both published evidence and established clinical practice play a part in decision-making regarding risk factors for pediatric dysphagia.

In Component B: “Oral Trials”, the inclusion of fluid trials reached near unanimous consensus amongst panelists (90% agreement) after the first eDelphi round (Table 3). In comparison, no agreement was reached between panelists to either include or exclude solid trials (46%) (Table 4). Interestingly, despite there being limited guidance in the literature as to screening protocols for children, this outcome aligns with many adult dysphagia screening tools [44,45,46] as well as recent research developing items for a novel screening tool post pediatric stroke [21]. Panelists agreed that bottle-feeding and sipper cup drinking were essential elements to include. In keeping with this, the COFS has three parts to its ‘oral trials’ component: breastfeeding, bottle-feeding, and sipper/open cup drinking to accommodate children of all developmental stages (Figure 1). Despite agreement to include these oral trial elements, there was a lack of consensus as to exactly which oral trial protocols should be included in the COFS. This uncertainty was likely due to the lack of published, validated oral trial screening protocols for this population in the literature. The 3-ounce Water Swallow Challenge is currently the only known oral trial protocol validated in children >2 years of age, who are sipper or open cup drinking [27,28]. Given this evidence, and the fact that it almost reached consensus (71% agreement) amongst panelists, this open/sipper cup drinking protocol was included in the COFS, alongside breast- and bottle-feeding protocols. Whilst the COFS represents the first known pediatric dysphagia screening tool with an oral trial component accommodating the full range of pediatric fluid intake methods, it is important to note that none of these protocols reached quantitative consensus amongst panelists, and their inclusion was driven by necessity. Development of valid and reliable pediatric oral trial protocols remains a critical area for further research.

Finally, in Component C: “Signs of Dysphagia”, there was again good agreement amongst panelists. Almost half the items reached consensus after the first round, with items that required a second round reaching high levels of agreement once reworded to ensure they were identifiable to nursing staff (Table 3). This tailoring is an important distinction between many current tools and the COFS, as the COFS is intended for nursing to complete based on observation of an oral trial protocol, rather than caregiver report alone. Potentially due to this reduced reliance on caregiver reporting, it is also one of the few tools that compiles multiple signs of dysphagia, rather than using predominantly coughing and choking. Whilst coughing and choking still reached consensus to include, items less frequently seen in other pediatric screening tools (e.g., wet vocalizations, color changes, new onset or increased wheezing or stridor) also reached consensus amongst panelists. Whilst the COFS doesn’t rely solely on caregiver report, child- and caregiver-reported pain/concerns were still seen as essential items to include within this component, likely to increase the reliability of the COFS across more than just a snapshot in time. Ultimately, from here, future large dataset research involving further psychometric testing of the COFS with large sample sizes across varying populations and ages is required to determine the validity and reliability of items within this COFS component.

Another finding in this study was the number of panelists who reported mandatory referral pathways for dysphagia assessment within their service. In the absence of current screening processes, mandatory referral pathways are used to ensure children at high risk for dysphagia are referred to a dysphagia specialist and assessed in a timely manner. If found to be valid and reliable, implementation of the COFS for all children may offer an alternative to mandatory pathways. This would ensure all children at risk get referred. However, for those not at risk, screening can help avoid over-referral to speech pathology and enable earlier resumption of oral feeding for those children without dysphagia.

There were several strengths to this study. The first was the international representation of panelists across a range of pediatric acute and critical care hospital settings and with a range of practice experiences. This wide sample supports the transferability of the proposed COFS tool, as well as the integration of new and existing knowledge amongst panelists, helping ensure the COFS is practical yet grounded in expert knowledge. Another key strength of this study was the high retention rate of panelists between rounds, indicating continued diversity of panelists, likely enhancing the validity of the results [37] and supporting the reproducibility of findings [47]. This high retention rate also likely indicates subject motivation amongst panelists [48]. However, several limitations to this eDelphi study are also acknowledged. Firstly, all panelists were speech pathologists, limiting content validity from a nursing perspective, which was provided indirectly by a nurse educator as part of the steering group. Despite international representation of panelists, this representation was primarily from first-world, English-speaking countries, potentially restricting generalizability to low-resource and non-English-speaking settings. In addition to this, the use of a modified two-round eDelphi, whilst enhancing retention, potentially reduced opportunities for panelists to reach consensus on items [36]. Panelists were presented with pre-generated items in the first round, potentially reducing innovation in item content [49]. Panelists were also not given the opportunity to re-rate items that achieved consensus in round one, potentially impacting their ability to gain a higher level of consensus [49]. Finally, while predefined consensus thresholds were used, some items were either retained or removed based on further qualitative feedback from panelists and steering group decision, potentially introducing some bias into the results [36].

Further research is now required to test the psychometric properties of the COFS (content validity, sensitivity/specificity, inter-rater reliability, and feasibility) in real-world acute and critical care hospital settings. Input from a range of end-users (i.e., nursing and medical staff, parents, and children) in addition to speech pathologists is now required to ensure the COFS is feasible and acceptable to all involved. Future implementation work should consider how differences in service structures, staffing models, and resource availability will likely impact uptake of the COFS, as well as whether nursing staff training on the COFS is required, and if so, exploring structured educational strategies (e.g., simulation-based training, potentially including virtual reality) [50].

## 5. Conclusions

This study has provided initial content validity for the items in a novel nurse-led pediatric dysphagia screening tool, the Children’s Oral Feeding Screener (COFS). This research represents an advance in early dysphagia screening for children 0–16 years admitted to pediatric acute and/or critical care hospital settings. Consensus was achieved amongst panelists and the steering group on key elements and items required in the screening tool. Further psychometric testing of the COFS in clinical settings is now required to establish the validity and reliability of this tool. Once fully developed, the COFS has the potential to adapt service pathways for pediatric dysphagia care.

## Figures and Tables

**Table 1:** Eligibility criteria for eDelphi panelists.

Inclusion Criteria	Exclusion Criteria
Speech pathologists or occupational therapists involved in the assessment and diagnosis of oropharyngeal dysphagia Minimum two years’ experience working with oropharyngeal dysphagia in pediatric acute and/or critical care settings Provided services to children with dysphagia on average at least weekly Worked in this setting within the last five years	Professionals other than speech pathologists or occupational therapists <two years’ clinical experience providing services to pediatric acute and/or critical care settings Only working in an adult setting Have not worked with children with dysphagia within an acute or critical care setting in the last five years Minimal exposure to oropharyngeal dysphagia presentations within workplace (e.g., predominantly seeing communication, fussy eating)

**Table 2:** Panelists’ demographics.

Demographic		Total Consented (n = 72) n (%)	eDelphi Round One (n = 50) n (%)	eDelphi Round Two (n = 41) n (%)
**Location:**				
Primary country of practice ^^^	Australia	32 (44%)	23 (46%)	19 (46%)
	UK	21 (29%)	17 (34%)	14 (34%)
	United States of America	13 (18%)	5 (10%)	4 (10%)
	New Zealand	7 (10%)	6 (12%)	5 (12%)
	South Africa	2 (3%)	2 (4%)	2 (5%)
	Hong Kong	1 (1%)	1 (2%)	1 (2%)
**Clinical Setting:**				
Setting ^^^	Public hospital	67 (93%)	6 (8%)	37 (90%)
	Private hospital	6 (8%)	45 (90%)	4 (10%)
Location ^^^	Metropolitan	61 (85%)	41 (82%)	33 (80%)
	Regional	19 (26%)	14 (28%)	13 (32%)
	Remote	2 (3%)	1 (2%)	1 (2%)
Hospital size	>300 beds	23 (32%)	12 (24%)	9 (22%)
	100–299 beds	24 (33%)	19 (38%)	15 (37%)
	50–99 beds	9 (13%)	6 (12%)	5 (12%)
	10–49 beds	15 (21%)	13 (26%)	12 (29%)
	<10 beds	1 (1%)	0 (0%)	0 (0%)
**Clinical Experience:**				
Profession	Speech Pathologist	72 (100%)	50 (100%)	41 (100%)
Additional qualifications ^^^	Graduate Certificate/Diploma	13 (18%)	10 (20%)	7 (17%)
	Masters	29 (40%)	21 (42%)	18 (44%)
	PhD Candidate	5 (7%)	4 (8%)	3 (7%)
	PhD	7 (10%)	3 (6%)	3 (7%)
	Other	4 (6%)	4 (8%)	4 (10%)
	None	24 (33%)	15 (3%)	12 (29%)
Experience *	2–3 years	15 (21%)	11 (22%)	8 (20%)
	4–5 years	9 (13%)	6 (12%)	6 (15%)
	6–10 years	9 (13%)	6 (12%)	5 (12%)
	>10 years	39 (54%)	27 (54%)	22 (54%)
Recency of practice *	Current	68 (94%)	47 (94%)	39 (95%)
	<1 year	3 (4%)	3 (6%)	2 (5%)
	<2 years	1 (1%)	0 (0%)	0 (0%)
Frequency of practice *	1–5 children/week	20 (28%)	16 (32%)	13 (32%)
	6–10 children/week	18 (25%)	12 (24%)	10 (24%)
	>10 children/week	34 (47%)	22 (44%)	18 (44%)
Experience ^^,^*	Acute wards	67 (93%)	47 (94%)	40 (98%)
	Emergency	22 (31%)	15 (30%)	14 (34%)
	NICU	52 (72%)	39 (78%)	32 (78%)
	PICU	50 (69%)	34 (68%)	26 (63%)
	Pediatric HDU	32 (44%)	24 (48%)	20 (49%)
PICU experience **	0 children/week	18 (25%)	13 (26%)	10 (24%)
	1–4 children/week	35 (49%)	26 (52%)	24 (59%)
	5–9 children/week	17 (24%)	10 (20%)	6 (15%)
	>10 children/week	2 (3%)	1 (2%)	1 (2%)

Note: PhD—Doctor of Philosophy; NICU—Neonatal intensive care unit; PICU—Paediatric intensive care unit; HDU—High dependency unit. ^^^ Panelists may be represented more than once. * Specific to the assessment and/or management of pediatric oropharyngeal dysphagia in acute and/or critical care hospital settings. ** Experience specific to assessment and/or management of pediatric oropharyngeal dysphagia in the pediatric intensive care unit (PICU).

**Table 3:** Agreement for all items included in the preliminary Children’s Oral Feeding Screener (COFS) tool.

COFS Component	Items	Round Included	Agreement to Include n (%)
**Red Flags** Presentations requiring immediate referral for a comprehensive swallow assessment by a dysphagia specialist	New tracheostomy	1	49 (98%)
	Bilateral vocal fold palsy	1	48 (96%)
	Inability to manage secretions	1	46 (92%)
	Absent cry/voice	1	45 (90%)
	Unilateral vocal fold palsy	1	43 (86%)
	*(Reworded)* Inability to protect airway (e.g., absent or diminished cough)	2	37 (90%)
	*(Reworded)* New onset facial asymmetry	2	33 (80%)
	*(New item)* Parent or nursing report of feeding and/or swallowing difficulties	2	36 (88%)
**Oral Trial Components** Nurse-led oral trial screening protocol/s	**Elements** Observation of fluids	1	45 (90%)
	Bottle feed where volume observed is set according to skill/usual volumes	1	38 (76%)
	Observation of usual sipper/open cup drinking	1	41 (82%)
	Protocols * (Reworded) Water swallow test—90 mL (1/2 cup) of single or consecutive sips of thin fluids (>2 years old) from sipper/open cup	2	29 (71%)
	* (Reworded) Breastfeed in usual position (observation of at least 3 min)	2	27 (66%)
	* (New item) Bottle feed in infants usual position using usual bottle or if not available hospital slow flow teat (observation of at least 3 min)	2	20 (49%)
**Signs of Dysphagia** Signs (identifiable to nursing staff), suggestive of oropharyngeal dysphagia in pediatrics, requiring immediate referral for a comprehensive swallow assessment by a dysphagia specialist	Wet/gurgly breathing	1	48 (96%)
	Wet vocalizations	1	46 (92%)
	Apnea with feeding	1	46 (92%)
	Color changes	1	46 (92%)
	Oxygen desaturations with feeding	1	43 (86%)
	Coughing	1	40 (80%)
	*(Reworded)* Increased respiratory rate/work of breathing outside of child’s typical ranges	2	39 (95%)
	*(Reworded)* New onset or increased wheezing whilst feeding	2	38 (93%)
	*(Reworded)* New onset or increased stridor whilst feeding	2	37 (90%)
	*(Reworded)* Fremitus/rattly chest during/after eating or drinking	2	36 (88%)
	*(Reworded)* Pooling of food/fluids in child’s mouth that is increased or unusual for them	2	34 (83%)
	*(New item)* Choking with feeding	2	41 (100%)
	*(New item)* Child reported pain, or differences to eating or drinking	2	36 (88%)
	*(New item)* Parental concern with feeding	2	34 (83%)

* These items did not meet the inclusion criteria (>75% agreement) but were later included based on steering group considerations of additional qualitative comments made by panelists.

**Table 4:** Agreement for all items excluded from the preliminary Children’s Oral Feeding Screener (COFS) tool.

COFS Component	Items		Round Excluded	Agreement to Include n (%)
**Red Flags** Presentations requiring immediate referral for a comprehensive swallow assessment by a dysphagia specialist	*(Moved into readiness section)* Unable to be positioned in bed safely		1	19 (38%)
	*(Moved into readiness section)* Ability to maintain alertness		1	21 (42%)
	* High flow nasal canula		2	33 (80%)
	New onset slurred speech		2	27 (66%)
	* (Reworded) New onset stridor		2	35 (85%)
	* (Reworded) Unable to move tongue in controlled way		2	31 (76%)
	*(Reworded)* New onset voice changes		2	30 (73%)
	*(Reworded)* Significant increase in work of breathing at rest		2	26 (63%)
	*(Reworded)* New onset language changes/word finding difficulties		2	20 (49%)
**Oral Trial Components** Nurse-led oral trial screening protocol/s	** Elements			
		Time limited breastfeed	1	19 (38%)
		Observation of full breastfeed	1	30 (60%)
		Time limited bottle feed	1	14 (28%)
		Bottle feed where volume observed is set according to age	1	7 (14%)
		Observation of full bottle feed	1	30 (60%)
		Time limited sipper/open cup drinking	1	11 (22%)
		Sipper/open cup where volumes observed is set according to age	1	6 (12%)
		Sipper/open cup where volume observed is set according to skill/usual volumes	1	31 (62%)
		Time limited solids feed	1	4 (8%)
		Solid feed where volume observed is set according to skill/usual volumes	1	29 (58%)
		Observation of full solid feed with usual diet	1	29 (58%)
		Observation of solids	2	19 (46%)
	**Protocols**			
		* (Reworded) Bottle feed in infants usual position using usual bottle or if not available hospital slow flow teat [39]	2	31 (76%)
		*(Reworded)* Breastfeed in usual position [39]	2	27 (66%)
		*(Reworded)* Timed bottle feed (20 min) using usual bottle or if not available hospital slow flow teat	2	15 (37%)
		*(Reworded)* Untimed small tastes (10 mL) of breastmilk or formula via usual bottle or hospital slow flow teat [40]	2	8 (20%)
		*(Reworded)* Untimed bottle feed of half of infant’s usual volume using usual bottle or if not available hospital slow flow teat [41]	2	8 (20%)
		*(Reworded)* Minimum of 5 x single or consecutive sips from sipper/open cup [42]	2	16 (39%)
		^ 5 × mouthfuls of usual diet	2	31 (76%)
		5 × mouthfuls of IDDSI level 4 puree	2	13 (32%)
**Signs of Dysphagia** Signs (identifiable to nursing staff), suggestive of oropharyngeal dysphagia in pediatrics, requiring immediate referral for a comprehensive swallow assessment by a dysphagia specialist	Unable to consume amounts as per protocol		2	16 (39%)
	More than 3 sucks/swallow for bottle feeding infants		2	13 (32%)
	Refusal to eat/drink		2	7 (17%)
	*(Reworded)* Spillage of food/fluids from child’s mouth that is increased or unusual for them		2	29 (71%)
	*(Reworded)* Decline in alertness during feeding that is new/unusual for the child		2	29 (71%)
	*(Reworded)* Increase in secretions during feeding		2	27 (66%)
	*(Reworded)* Throat clearing during feeding		2	17 (41%)
	*(Reworded)* Gagging whilst feeding		2	12 (29%)
	* (New item) Distress cues during oral feeding (e.g., turning head away, clamping mouth shut, crying) that is unusual and persistent for the child		2	33 (80%)
	*(New item)* Teary eyes during feeding		2	22 (54%)

* These items met the inclusion criteria (>75% agreement) but were later excluded based on steering group considerations of additional qualitative comments made by panelists. ** These items were removed from round two, as were oral trial elements, designed to elicit what panelists felt were important components when creating oral trial protocols for round two. ^ This item met inclusion criteria (>75% agreement) but was later excluded as panelists did not reach agreement that solid trials in general should be included in the COFS.

## Data Availability

The author confirms that all data generated or analyzed during this study are included in this published article and within online supplementary resources. Furthermore, primary and secondary sources and data supporting the findings of this study were all publicly available at the time of submission.

## Abbreviations

The following abbreviations are used in this manuscript:

COFS	Children’s Oral Feeding Screener
COSMIN	Consensus-based Standards for the Selection of Health Measurement Instruments
DELPHISTAR	Delphi studies in social and health sciences—recommendations for an interdisciplinary standardized reporting
PICU	Pediatric intensive care unit

## Appendix A

**Table A1:** Delphi studies in social and health sciences—recommendations for an interdisciplinary standardized reporting (DELPHISTAR).

Topic	Section	Item	Checklist Item	Location Where Item is Reported
**I** **Title and Abstract**		1	Identification as a Delphi procedure in the title	Title
		2	Identification as a Delphi procedure in the abstract	Abstract
		3	Structured abstract	Abstract
**II** **Context**	**Formal**	4	Information about the sources of funding	End of manuscript
		5	Information about the team of authors and/or researchers (e.g., discipline, institution)	Title page/methods
		6	Information about method consulting	Methods
		7	Information about the project background	Introduction
		8	Information about the study protocol	Methods
	**Content**	9	Justification of the chosen method (Delphi procedure) to answer the research question	Methods
		10	Aim of the Delphi procedure (e.g., consensus, forecasting)	Introduction
**III** **Method**	**Body and Integration of knowledge**	11	Identification and elucidation of relevant expertise, spheres of experience, and perspectives (e.g., theory, practice, affected groups, disciplines)	Methods
		12	Handling of knowledge, expertise and perspectives which are missing or have been deliberately not integrated	N/a
		13	Basic definition of expert ^1^	Methods
	**Delphi variations**	14	Identification of the type of Delphi procedure and potential modifications (e.g., classic Delphi, real-time Delphi, group Delphi)	Methods
		15	Justification of the Delphi variation and modifications, including during the Delphi process, if applicable	Methods
	**Sample of experts**	16	Selection criteria for the experts (per round if there are different expert groups)	Methods
		17	Identification of the experts	Methods
		18	Information about recruiting and any subsequent recruiting of experts	Methods
	**Survey**	19	Elucidation of the content development for the questionnaire ^2^	Methods
		20	Description of the questionnaire (content and structure)	Methods
	**Delphi rounds**	21	Number of Delphi rounds	Methods
		22	Information about the aims of the individual Delphi rounds	Methods
		23	Disclosure and justification of the criterion for discontinuation	Methods
	**Feedback**	24	Information about what data was reported back per round	Methods
		25	Information on how the results of the previous Delphi round were fed back to the experts surveyed (e.g., via frequencies, mean values, measures of dispersion, listing of comments)	Methods
		26	Information on whether feedback was differentiated by specific groups (e.g., by field of expertise, institutional affiliation)	Methods
		27	Information about how dissent and unclear results were handled	Methods
	**Data analysis**	28	Disclosure of the quantitative and qualitative analytical strategy	Methods
		29	Definition and measurement of consensus	Methods
		30	Information on group-specific analysis or weighting of experts (e.g., theory vs. practice, discipline-specific analysis)	N/a
**IV** **Results**	**Delphi process**	31	Illustration of the Delphi process (e.g., in a flow chart)	Figure 2
		32	Information about special aspects during the Delphi process (e.g., deviations from the intended approach with justification)	Methods
		33	Number of experts per round (both invited and participating)	Results
	**Results**	34	Presentation of the results for each Delphi round and the final results	Results
**V Discussion**	**Quality of findings**	35	Highlighting the findings from the Delphi study	Discussion
		36	Validity of the results (e.g., transferability of the findings)	Discussion
		37	Reliability of the results (e.g., split half, inter-rater reliability)	Discussion
		38	Reflection on potential limitations (e.g., distortion, skewing, bias)	Discussion

^1^ “Experts” are the participants; this can be people from academia, practice, or representatives of lived experience (e.g., patients, family members). ^2^ The term “questionnaire” stands for the survey instrument regardless of whether quantitative or qualitative items are integrated or weighted.
